# A novel thymoma-associated autoimmune disease: Anti-PIT-1 antibody syndrome

**DOI:** 10.1038/srep43060

**Published:** 2017-02-20

**Authors:** Hironori Bando, Genzo Iguchi, Yasuhiko Okimura, Yukiko Odake, Kenichi Yoshida, Ryusaku Matsumoto, Kentaro Suda, Hitoshi Nishizawa, Hidenori Fukuoka, Atsuko Mokubo, Katsuyoshi Tojo, Yoshimasa Maniwa, Wataru Ogawa, Yutaka Takahashi

**Affiliations:** 1 Division of Diabetes and Endocrinology, Department of Internal Medicine, Kobe University Graduate School of Medicine, Kobe, Japan; 2 Division of Diabetes and Endocrinology, Kobe University Hospital, Kobe, Japan; 3 Department of Nutrition and Food Science, Kobe Women’s University Graduate School of Life Sciences, Kobe, Japan; 4 Mokubo Clinic, Kawasaki, Japan; 5 Division of Diabetes and Endocrinology, Department of Medicine, Jikei University School of Medicine, Tokyo, Japan; 6 Department of General Thoracic Surgery, Kobe University Graduate School of Medicine, Kobe, Japan

## Abstract

Anti-PIT-1 antibody syndrome has recently been reported and characterized by acquired growth hormone (GH), prolactin (PRL), and thyroid-stimulating hormone (TSH) deficiencies associated with autoimmunity to a pituitary specific transcription factor PIT-1, which plays an essential role in GH-, PRL-, and TSH-producing cells. Although circulating anti-PIT-1 antibody and PIT-1-reactive cytotoxic T cells (CTLs) were detected in the patients, the pathophysiology and precise mechanisms for the autoimmunity remain unclarified. During the follow up, thymoma was diagnosed in all 3 cases with anti-PIT-1 antibody syndrome. Immunohistochemical analysis revealed that PIT-1 was strongly expressed in neoplastic cortical thymic epithelial cells. Importantly, after thymectomy, the titer of anti-PIT-1 antibody decreased and reactivity of CTLs toward PIT-1 diminished. These data strongly suggest that the aberrant expression of PIT-1 in the thymoma plays a causal role in the development of this syndrome. Thus, we define that this syndrome is a novel thymoma-associated autoimmune disease.

The clinical symptoms of hypopituitarism are usually unspecific but it can cause life-threatening events, lead to increased mortality, and impair quality of life^1^; thus clinicians need to pay attention as a differential diagnosis and to perform replacement therapy as appropriate^2^,^3^. Various conditions in the hypothalamus and pituitary including tumor, surgery, irradiation, inflammation and autoimmune-related diseases are known to cause acquired hypopituitarism^4^. In particular, autoimmunity against pituitary gland is involved in the development of lymphocytic hypophysitis and isolated ACTH deficiency^5^,^6^,^7^.

The pituitary-specific transcriptional factor-1 (PIT-1, also known as POU1F1) is a member of the Pit-Oct-Unc (POU) homeodomain family that plays an essential role in the differentiation of somatotrophs, lactotrophs, and thyrotrophs in the anterior pituitary^8^. It also regulates the expression of growth hormone (GH), prolactin (PRL), and thyroid-stimulating hormone (TSH) and mutations in *PIT-1* gene cause congenital GH, PRL, and TSH deficiencies^9^.

We previously reported a novel clinical entity named anti-PIT-1 antibody syndrome, which is caused by autoimmunity against PIT-1 protein^10^. This syndrome is characterized by an acquired combined pituitary hormone deficiency exhibiting a specific defect in GH, PRL, and TSH, and a presence of circulating anti-PIT-1 antibody^10^,^11^. Thus far, 3 patients have been reported, in which multiple endocrine organs were involved in the autoimmunity such as thyroiditis, insulitis, and adrenalitis with autoantibodies in a various degree depending on the patient^8^. It indicated that this syndrome met the definition of autoimmune polyglandular syndrome (APS)^12^. As the underlying mechanisms, it has been reported that cytotoxic T cells (CTLs) that react against the PIT-1 protein play a pivotal role in the development of this syndrome^13^. However, the underlying cause of the breakdown in immune tolerance for PIT-1 has not been clarified.

Thymus is a primary lymphoid organ, where T cells are differentiated. Positive and negative selection of T cells takes place in the thymus, which ensures the acquisition of central T cell tolerance^14^. Cortical thymic epithelial cells and their MHC antigen expression dictate positive selection, while medullary thymic epithelial cells that express autoantigens contribute to the negative selection^15^. It is well known that thymoma is closely associated with several autoimmune diseases such as myasthenia gravis (MG). Although the precise mechanisms remain unknown, it has been suggested that the aberrant expression of the antigen, acetylcholine receptor (AChR) in the tumor cells and a defect in the negative selection in thymoma may play a role in the development of autoimmunity^16^,^17^,^18^.

In this report, we demonstrate that patients with anti-PIT-1 antibody syndrome present with a thymoma and show substantial evidences that the thymoma plays a crucial role in the development of this disease.

The clinical characteristics of the 3 patients with anti-PIT-1 antibody syndrome were previously described in detail^8^ and described briefly as follows: Patient 1. A 44-year-old man without growth and developmental delay who presented with facial, finger, and arm edema as a result of central hypothyroidism. Endocrinological provocative test revealed that the secretion of GH and PRL were completely blunted, and that of TSH was severely impaired. Patient 2. A 75-year-old man with a history of slowly progressive insulin-dependent diabetes mellitus showed central hypothyroidism. The anterior pituitary function was in level to what was observed in patient 1. Autopsy and histological analysis was performed. Patient 3. A 78-year-old man showed acquired central hypothyroidism. The anterior pituitary function was similar in level to what was observed in both patients 1 and 2. Circulating anti-PIT-1 antibody was detected in all these patients.

## Results

### Thymomas were diagnosed in all patients with anti-PIT-1 antibody syndrome

**Figure 1:**
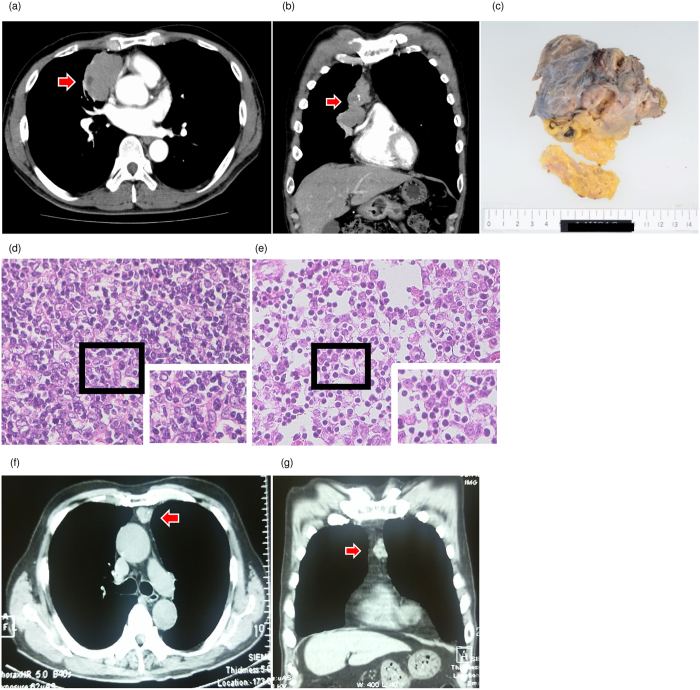
The findings of thymomas. (**a**,**b**) Computed tomography (CT) findings of thymomas of Patient 1. (**a**) Horizontal plane, (**b**) Coronal plane. Arrows indicate thymoma. (**c**) Macroscopic finding of the thymoma in patient 1. (**d**,**e**) Hematoxylin and eosin stain of thymoma tissues. (**d**) Patient 1, (**e**) Patient 2 (×400). (**f**,**g**) CT findings of the thymoma of patient 3. (**f**) Horizontal plane, (**g**) Coronal plane. Arrows indicate thymoma.

During the follow up, a mediastinal tumor was detected by checkup chest X-ray and subsequent computed tomography (CT) imaging confirmed the diagnosis in patient 1 (Fig. 1a and b). The tumor was resected and histological analysis revealed a diagnosis of type B2 thymoma (Fig. 1c and d). The patient did not undergo any immunotherapy including steroids during the clinical course. In patient 2, we examined the autopsy specimen and found a type AB thymoma (Fig. 1e). It is characterized by a dense infiltration of lymphocytes in these tumors. In addition, chest CT revealed an anterior mediastinal tumor with a suspicion of thymoma in patient 3 (Fig. 1f and g). Because of the advanced age, the tumor has been carefully observed in this patient. These data indicate that although one case has not been histologically proven, all the patients with anti-PIT-1 antibody syndrome were associated with thymomas, suggesting that thymoma plays a role in the pathogenesis of this syndrome.

### Aberrant expression of PIT-1 protein in the neoplastic cortical thymic epithelial cells

**Figure 2:**
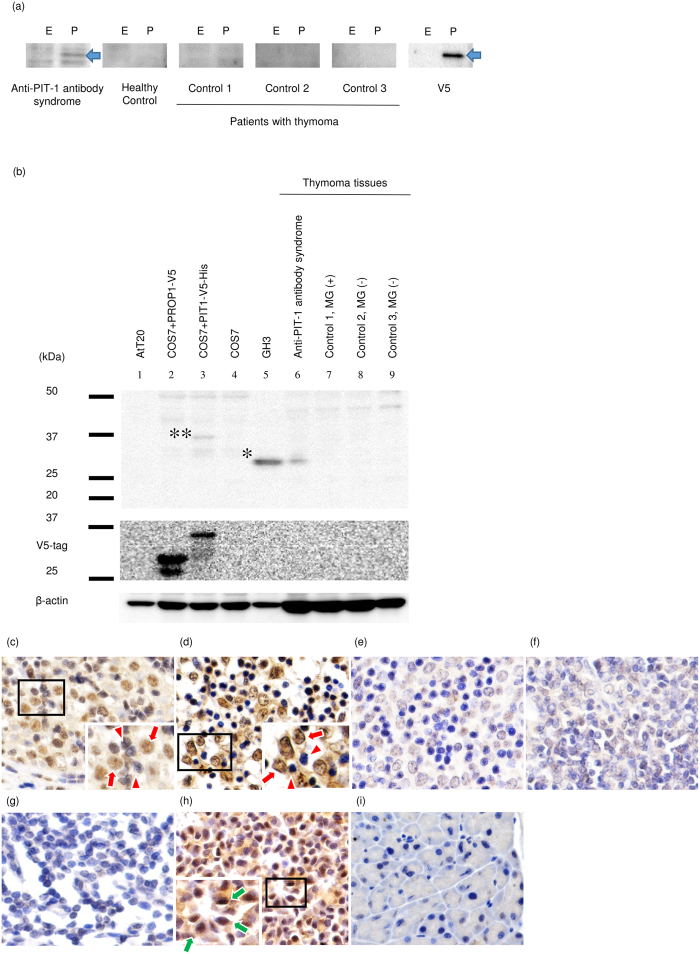
PIT-1 protein was aberrantly expressed in neoplastic thymic epithelial cells of the patients with anti-PIT-1 antibody syndrome. (**a**) Western-blotting analysis for the detection of anti-PIT-1 antibody in the serum of patients with thymoma. E and P indicate empty vector (control) or PIT-1 expressing vector, which were transfected into COS7 cells, respectively. Arrows indicate V5-tagged PIT-1 protein. Sera were diluted 500-folds. (**b**) Western-blotting analysis using the lysate from thymoma tissues. PIT-1 protein was detected in the thymoma tissue of patient with anti-PIT-1 antibody syndrome (lane 6). In contrast, PIT-1 protein was not detected in the thymoma tissues from patients with MG (B2 type, lane 7) or without MG (B2 type, lane 8 and AB type, lane 9). *and **indicate endogenous PIT-1 in GH3 cells and V5-tagged PIT-1 protein in PIT-1-expressing COS7 cells, respectively. (**c**–**g**) Immunohistochemical analysis of thymoma tissues. (**c**) Patient 1, (**d**) Patient 2, (**e**) B2 type thymoma with MG, (**f**) B2 type thymoma without MG, (**g**) normal thymic tissue attached to thymoma with MG ( × 400), (**h**) mouse pituitary tissue (positive control), and (**i**) mouse pancreas tissue (negative control). PIT-1 protein was detected in neoplastic thymic epithelial cells (red arrow) not in lymphocytes in the 2 cases examined (red arrowhead). Green arrows indicate the PIT-1 positive cells in the mouse pituitary.

It is well known that thymoma is causally associated with several autoimmune diseases such as MG. Because acquired immune intolerance to PIT-1 plays a pivotal role in the development of anti-PIT-1 antibody syndrome^10^,^13^,^19^, we hypothesized that PIT-1-reactive T cells may be positively selected in the thymoma tissues. Since it has been reported that the aberrant expression of AChR is associated with autoimmunity to AChR in MG^20^, we analyzed the expression of PIT-1 protein in the thymoma tissue. First, we verified that the serum of patients with thymoma without hypopituitarism did not have anti-PIT-1 antibody (Table 1, Fig. 2a). It indicated that anti-PIT-1 antibody is specific for the patients of anti-PIT-1 antibody syndrome. Intriguingly, immunoblotting analysis demonstrated a substantial expression of PIT-1 protein in the thymoma of patient 1 (Fig. 2b, lane 6). In contrast, PIT-1 protein was not detected in the thymoma tissues from patients with MG or without MG (Fig. 2b, lane 7–9). Furthermore, immunohistochemical analysis showed an aberrant expression of PIT-1 in the neoplastic cortical thymic epithelial cells in patient 1 (Fig. 2c) and 2 (Fig. 2d) in contrast to those cells in thymoma with (Fig. 2e) or without MG (Fig. 2f), or thymus cells (Fig. 2g).

### Thymectomy drastically improved the immune response to PIT-1

**Figure 3:**
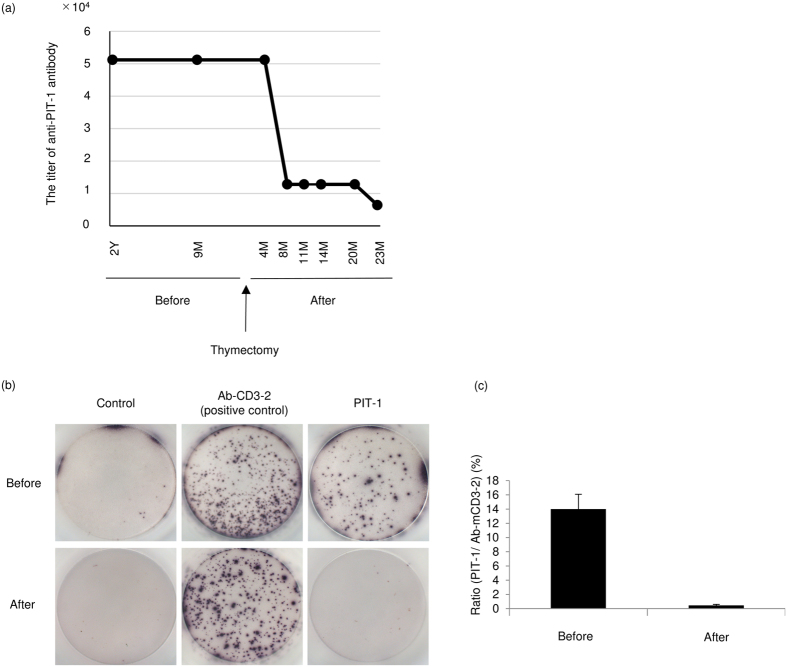
The changes in the titer of serum anti-PIT-1 antibody and PIT-1 reactive CTLs after thymectomy in Patient 1. (**a**) the changes in the titer of anti-PIT-1 antibody. The titer was determined by immunoblotting with a serial dilution of sera. The results of immunoblotting are shown in Supplementary Figure. (**b**,**c**) (**b**) Images of the results of enzyme-linked immunospot (ELISpot) assay. (**c**) Quantitative analysis of the ratio of the spots toward PIT-1 in accordance with positive control.

We examined the titer of anti-PIT-1 antibody by serial dilution using preserved sera before and after thymectomy in patient 1 (Fig. 3a and Supplementary Figure). Intriguingly, before thymectomy, the titer remained unchanged; however, after thymectomy the titer decreased by one eighth during the following 2 years. We further evaluated the peripheral CTLs reacted with PIT-1 protein before and after thymectomy. Convincingly, CTLs reacted with PIT-1 protein, as detected by ELISpot assay, diminished after thymectomy (Fig. 3b and c).

## Discussion

In this report, we have demonstrated that thymoma was associated with all patients with anti-PIT-1 antibody syndrome. PIT-1 was highly expressed in the neoplastic cortical thymic epithelial cells in the two cases examined. Importantly, after thymectomy, the titer of anti-PIT-1 antibody substantially decreased and specific CTLs for PIT-1 diminished in one patient who underwent surgery. These data strongly suggest that thymoma that aberrantly expressed PIT-1 plays an essential role in the development of this disease.

It is well known that thymoma is closely associated with the pathogenesis of MG. Thymomas are histologically classified into types A, AB, B1, B2, and B3 (WHO classification)^21^. It has been reported that autoimmune diseases are associated particularly with types AB and B^22^, as observed with our cases. Approximately 10% of patients with autoimmune MG have a thymoma, which plays a role in disease initiation through multiple mechanisms including the expression of self-antigens such as AChR and impaired negative selection of autoreactive T cells^23^. In anti-PIT-1 antibody syndrome, it is speculated that the aberrant expression of PIT-1 evoked positive selection for PIT-1-reactive T cells and the defect in negative selection in thymoma tissue induced breakdown in immune tolerance for PIT-1.

One of the key questions is the reason for the aberrant expression of PIT-1 in the thymoma. Recently, it has been reported that mutations of epigenetic regulatory genes are common in thymic neoplasms^24^, suggesting that epigenetic changes such as histone modification, chromatin remodeling, and DNA methylation pathways may occur and cause a dysregulated expression of silencing gene in thymoma. It is possible that such epigenetic changes cause the aberrant expression of PIT-1 in the thymoma tissue of anti-PIT-1 antibody syndrome. It has also been reported that PIT-1 was aberrantly expressed in some tumor tissues, such as breast cancer and acute myeloid leukemia cells, and plays an important role in the carcinogenesis, promotion of tumor growth and metastasis^25^,^26^. Although the mechanisms, in which PIT-1 was aberrantly expressed in the breast cancer have not been elucidated, it has been reported that deregulation of several embryonic transcription factors including POU homeobox genes were expressed at high levels in human breast cancer^27^,^28^,^29^. Similar mechanisms might be present in the thymoma tissue of anti-PIT-1 antibody syndrome.

In MG, the anti-AChR antibody is directly responsible for the pathogenesis^23^,^30^. Anti-AChR antibodies are the most common type of the pathogenesis of MG. Anti-AChR antibody activates the classical complement pathway, formation of the membrane attack complex initiated by activated C3, causes severe structural injury of endplates and lyses the postsynaptic membrane^31^. We examined the pathogenic role of anti-PIT-1 antibody in detail but we could not find any effects^13^, suggesting that this antibody is only a marker of this syndrome. On the other hand, PIT-1-reactive CTLs plays an essential role in the development of the disease^13^. In MG, it has been speculated that the first alteration in intratumorous T cell thymopoiesis may involve the generation of muscle-specific CTLs, and the resulting muscle damage could lead to various autoimmune responses including a production of autoantibodies against AChR and intracellular antigens^32^. It is speculated that anti-PIT-1 antibody may be produced after the injury of PIT-1 expressing cells by CTL and the resulting exposure of the nuclear protein PIT-1.

Interestingly, not only did the titer of anti-PIT-1 antibody decrease but peripheral PIT-1-reactive CTLs also diminished after thymectomy without any immunosuppressive therapy, suggesting that the thymoma played an essential role in the breakdown of immune tolerance for PIT-1. In MG, disease remission or improvement is expected following thymectomy^33^. In the patients with anti-PIT-1 antibody syndrome who underwent thymectomy, although the titer of antibody and PIT-1-reactive CTLs decreased, we could not observe an improvement of anterior pituitary function at two years after thymectomy (Table 2). As shown in the autopsy case, GH-, PRL-, TSH-, and PIT-1-positive cells were absent, with inflammation and marked fibrosis observed in the anterior pituitary^10^. This suggests that when hormone secretion was impaired, target cells might already be irreversibly injured by CTLs. However, because an increase in remission rates over 7 to 10 years has been reported in MG^34^, it is necessary to follow anterior pituitary function in the patients over the long term. It has been reported that some thymoma patients may have impaired anterior pituitary function; however, detailed examination has not been performed^35^. Although the prevalence of anti-PIT-1 antibody syndrome in patients with thymoma is unknown, it is warranted to screen pituitary function in patients with thymoma especially presenting with symptoms related to hypothyroidism.

It is also known that thymoma is associated with paraneoplastic syndrome, especially paraneoplastic neurological syndrome such as paraneoplastic encephalomyelitis and paraneoplastic limbic encephalitis^36^,^37^,^38^. Most of the patients reveal autoantibodies against the common antigens between the tumor and central nervous system. Some of the antibodies play a pathogenic role in the development of disease and the resection of tumor is efficacious. In these aspects, it is considerable that anti-PIT-1 antibody syndrome is a paraneoplastic syndrome. It is speculated not only thymoma but also other tumors with an aberrant expression of PIT-1 protein may cause this syndrome.

In conclusion, we define anti-PIT-1 antibody syndrome as a thymoma-associated autoimmune disease, which exhibits an acquired and specific GH, PRL, and TSH deficiency.

## Methods

### Patients

This study was approved by the ethics committee of Kobe University Graduate School of Medicine and all methods were performed in accordance with the guidelines. The patients provided a written informed consent for the analysis.

Titration analysis of anti-PIT-1 antibody, ELISpot assay, and western-blotting analysis were performed using samples from patient 1. Immunohistochemical analysis of thymoma tissues were performed in both patients 1 and 2. In patient 3, the tumor has not been resected and has carefully been observed.

The clinical characteristics of control patients with thymoma without anti-PIT-1 antibody syndrome were shown in Table 1.

### Animals

Mouse experiments were performed according to the guidelines of the Animal Ethics Committee of Kobe University Graduate School of Medicine. The experimental protocols were approved by the Institutional Animal Care and Use Committee and carried out according to the Kobe University Animal Experiment Regulations. The C57BL/6 mice were kept on a 12-h day/night cycle and had ad lib access to water and normal chow. The pituitary and pancreas tissues were used as controls for immunohistochemical analysis.

### Cell culture and transfection experiment

COS7, GH3, and AtT20 cells were obtained from ATCC. These cells were cultured in Dulbecco’s modified eagle medium containing 10% fatal calf serum. V5 and His tagged human PIT-1 expression vector (pcDNA3.1D/V5-His-hPIT-1) and V5-tagged human PROP-1 expression vector (pcDNA3.1/V5-hPROP1) were constructed using pcDNA3.1 Directional TOPO expression kit and pcDNA3.1/V5 TOPO TA Expression Kit, respectively (Invitrogen, Carlsbad, CA). These plasmids were transiently transfected into COS7 cells using X-treamGENE HP DNA Transfection Reagent (Roche, Indianapolis, IN).

### Immunoblotting analysis

For the preparation of lysates, tissue samples and cultured cell lines were dissolved in RIPA buffer [20 mM Tris⋅HCl, pH 7.4/150 mM NaCl/1% (v/v) Nonidet P-40/0.1% (w/v) SDS] containing 1% protease inhibitor cocktail (Nacalai Tesque, Kyoto, Japan) and cleared of debris by centrifugation at 15,000 × g. For the titration analysis of anti-PIT-1 antibody, 10 μg of proteins were loaded per lane. Patient sera were used as primary antibody at the indicated dilutions. HRP-conjugated goat anti-human IgG + A + M (H + L)-HRP (1:50000; Invitrogen) was used as secondary antibody. Anti-PIT-1 antibody (1:200; Santa Cruz Biotechnology, Santa Cruz, CA), anti-β-actin (Sigma-Aldrich, St Louis, Mo) and anti-V5 antibody (Invitrogen) were used as primary antibody. HRP-conjugated anti-rabbit IgG (1:5000; Invitrogen) or HRP-conjugated anti-mouse IgG (1:5000; Invitrogen) was used as secondary antibody. Signals were detected with a Chemilumi-One chemiluminescence kit (Nacalai Tesque) and an image analyzer (ImageQuant LAS 4000, GE Healthcare Life Science).

### Enzyme-Linked Immunospot (ELISpot) assay

ELISpot assays were performed according to the manufacture’s instruction as previously described^11^. Briefly, aliquots of 2.0 × 10^5^ isolated lymphocytes from peripheral blood per well were incubated with recombinant PIT-1 protein (10 μg/mL, Santa Cruz Biotechnology) in 96-well microtiter plates pre-coated with anti-human IFN-γ antibody for 48 hours (ELISpot^PRO^ for Human IFN-γ; Mabtech, Stockholm, Sweden). After washing, biotin-conjugated anti-cytokine antibody (7-B6-1) was added and incubated followed by streptavidin conjugated with alkaline phosphatase. Finally, 5-bromo-4-chloro-3-indolyl-phosphate/nitro blue tetrazolium substrate solution was added and incubated for 48 hours. Stimulation by Ab-mCD3-2 was used as a positive control. All assays were performed with quadruplicate for each antigen. The spots were counted viewing through a microscope by 2 independent investigators in a blinded way. Homogeneously stained spots were evaluated as positive.

### Immunohistochemical analysis of thymoma tissues

The tissue specimens were fixed in 10% buffered formaldehyde, dehydrated in graded ethanol, and embedded in paraffin. PIT-1 immunostaining was performed with anti-PIT-1 antibody (1:75; Santa Cruz Biotechnology) using Can Get Signal immunostain (TOYOBO, Osaka, Japan). EnVision^+^ Single Reagent (Dako, Glostrup, Denmark) was used for visualization. Images were obtained with a BZ-8100 microscope (Keyence, Osaka, Japan).

## Additional Information

**How to cite this article**: Bando, H. *et al*. A novel thymoma-associated autoimmune disease: Anti-PIT-1 antibody syndrome. *Sci. Rep.* **7**, 43060; doi: 10.1038/srep43060 (2017).

**Publisher's note:** Springer Nature remains neutral with regard to jurisdictional claims in published maps and institutional affiliations.

## Supplementary Material

Supplementary Figure

## Figures and Tables

**Table 1:** The clinical characteristics of control patients with thymoma without hypopituitarism.

	Control 1	Control 2	Control 3
Sex	Female	Male	Male
Age	62	83	58
Histological classification of thymoma	B2	B2	AB
Myathenia Gravis	(+)	(−)	(−)
Other autoimmune diseases	(−)	(−)	(−)
Immunotherapy during the clinical course	Predonisolone	(−)	(−)

**Table 2:** Labolatory data before and after thymectomy.

	Before thymectomy	After thymectomy
White blood cell (/μL)	4400	5100
Red blood cell (10^4^/μL)	519	448
Hemoglobin (g/dL)	15.9	12.9
Hematocrit (%)	46.0	38.6
Platelet (10^4^/μL)	22.1	24.2
Aspartate aminotransferase (U/L)	24	25
Alanine aminotransferase (U/L)	18	18
γ-glutamyltransferase (U/L)	50	56
Alkaline phosphatase (U/L)	298	445
Lactate Dehydrogenase (U/L)	185	156
Creatine phosphokinase (U/L)	121	67
Choline esterase (U/L)	330	320
Sodium (mEq/L)	142	141
Potassium (mEq/L)	4.1	4.0
Chloride (mEq/L)	107	104
Urea nitrogen (mg/dL)	8.8	9.3
Creatinine (mg/dL)	0.75	0.84
Total protein (g/dL)	7.5	7.4
Albumin (g/dL)	4.7	4.2
Total bilirubin (mg/dL)	0.7	0.6
Anti-acetylcholine receptor antibody	(−)	(−)
Basal GH (ng/mL)	<0.2	<0.1
Peak GH (ng/mL)	<0.2	<0.1
Basal PRL (ng/mL)	<0.3	<0.6
Peak PRL (ng/mL)	<0.3	<0.6
Basal TSH (IU/mL)	0.032	<0.004
Peak TSH (IU/mL)	0.037	<0.004
Basal ACTH (pg/mL)	35.2	30.3
Peak ACTH (pg/mL)	162	89.9
Basal Cortisol (μg/dL)	2.7	11.0
Peak Cortisol (μg/dL)	17.8	17.5
Basal LH (mIU/mL)	2.4	1.7
Peak LH (mIU/mL)	9.8	21.4
Basal FSH (mIU/mL)	5.3	9.1
Peak FSH (mIU/mL)	17.8	17.8
FT4 (ng/dL)*	1.19	1.05
Testosterone (ng/mL)	5.2	5.1
Insulin-like growth factor 1 (ng/mL)*	151	133
Anti-thyroglobulin antibody (IU/mL)	796	577

GH; Growth hormone, PRL; Prolactin, TSH; Thyroid stimulating hormone.

ACTH; Adrenocorticotropic hormone, LH; Lutenizing hormone, FSH; Follicle stimulating hormone.

*Under replacement of rhGH and levothyroxine. A part of the data was described previously (ref. 10).
