# Supplementary material for: A novel thymoma-associated autoimmune disease: Anti-PIT-1 antibody syndrome
Source: Sci Rep. 2017 Feb 20;7:43060. doi: 10.1038/srep43060 (PMC5316939; doi:10.1038/srep43060)

A novel thymoma-associated autoimmune disease: Anti-PIT-1 antibody syndrome

Hironori Bando[1], Genzo Iguchi[2], Yasuhiko Okimura[3], Yukiko Odake[1], Kenichi Yoshida[1], Ryusaku Matsumoto[1], Kentaro Suda[1], Hitoshi Nishizawa[2], Hidenori Fukuoka[2], Atsuko Mokubo[4], Katsuyoshi Tojo[5], Yoshimasa Maniwa[6], Wataru Ogawa[1], and Yutaka Takahashi[1*]

1. Division of Diabetes and Endocrinology, Department of Internal Medicine, Kobe University Graduate School of Medicine, Kobe, Japan

2. Division of Diabetes and Endocrinology, Kobe University Hospital, Kobe, Japan

3. Department of Nutrition and Food Science, Kobe Women's University Graduate School of Life Sciences, Kobe, Japan

4. Mokubo Clinic, Kawasaki, Japan

5. Division of Diabetes and Endocrinology, Department of Medicine, Jikei University School of Medicine, Tokyo, Japan

6. Department of General Thoracic Surgery, Kobe University Graduate School of Medicine, Kobe, Japan

Supplementary Figure. Titration analysis of anti-PIT-1 antibody.

The titer was determined by immunoblotting with a serial dilution of sera before and after thymectomy. The results of immunoblotting are shown. Arrows indicate the highest dilution of sera for detecting PIT-1 protein. E and P indicate empty vector (control) or PIT-1 expressing vector, which was expressed in COS7 cells, respectively.

Supplementary Figure

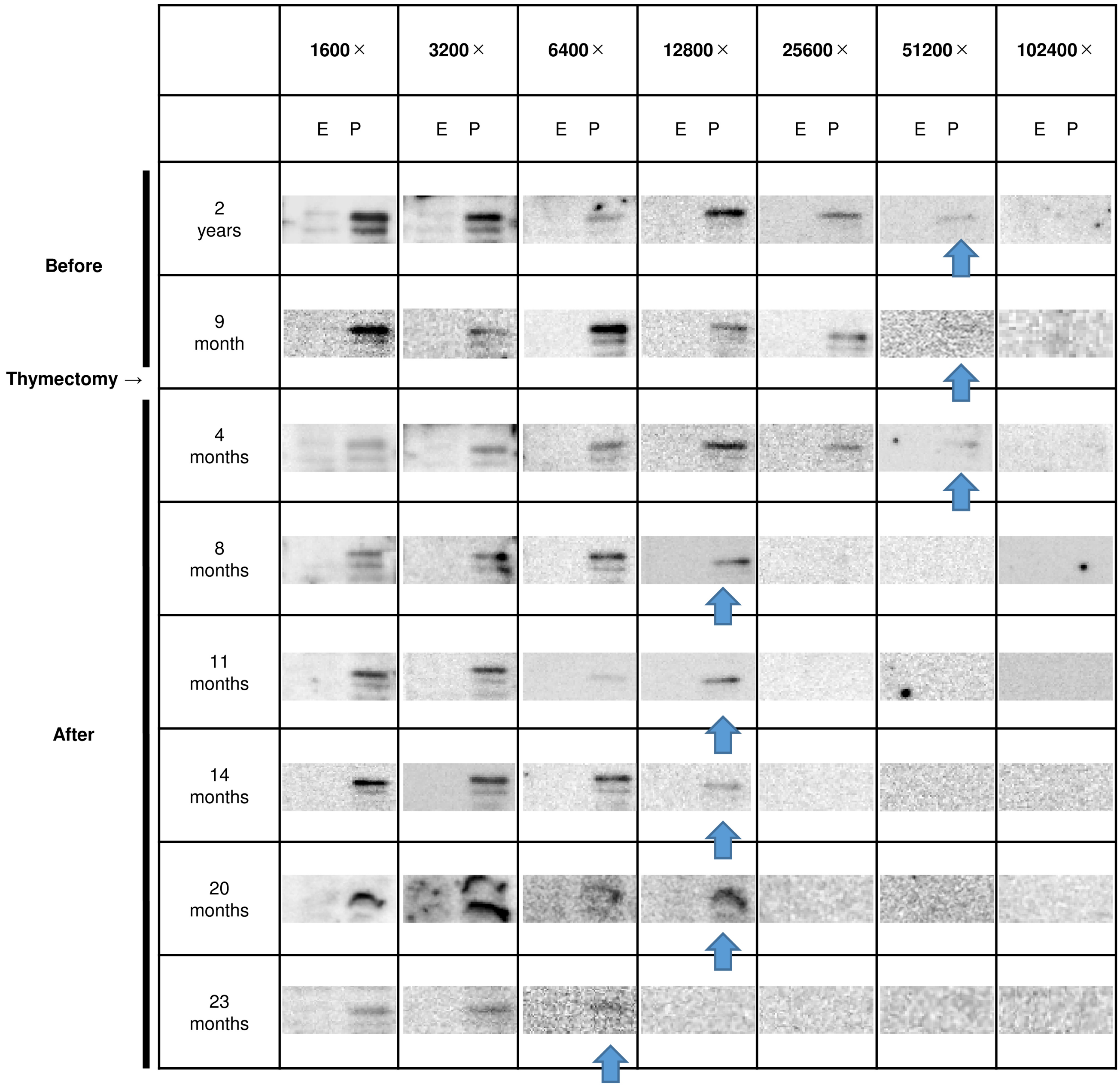

Supplement: Supplementary Figure [file srep43060-s1.pdf]
